# Medical Resource Use and Costs of Treating Sickle Cell-related Vaso-occlusive Crisis Episodes: A Retrospective Claims Study

**DOI:** 10.36469/jheor.2020.12852

**Published:** 2020-06-15

**Authors:** Nirmish Shah, Menaka Bhor, Lin Xie, Jincy Paulose, Huseyin Yuce

**Affiliations:** 1 Duke University, Durham, NC, USA; 2 Novartis Pharmaceuticals Corporation, East Hanover, NJ, USA; 3 SIMR, Inc., Ann Arbor, MI, USA; 4 New York City College of Technology, CUNY, Brooklyn, NY, USA

**Keywords:** sickle cell disease, vaso-occlusive crisis, hydroxyurea, Medicaid, economic outcomes

## Abstract

**Background:**

The study investigated the economic burden of vaso-occlusive crisis (VOC) among sickle cell disease (SCD) patients, through assessment of overall utilization and costs and costs per VOC episode (regarding the number of VOC episodes and health care setting, respectively).

**Methods:**

Using the Medicaid Analytic Extracts database, the first SCD-related diagnosis claim (index claim) between June 1, 2009–December 31, 2012 was identified among eligible adults. Patients were required to have continuous medical and pharmacy benefits for 6 months pre- and 12 months post-index. Discrete VOC claims identified within a 3-day gap were combined as a single VOC episode. Annual all-cause and SCD-related medical resources and costs were identified and stratified by number of VOC episodes during the 1-year follow-up period. Health care costs per VOC episode were also examined, stratified by care setting.

**Results:**

Enrollees included 8521 eligible patients with a mean age of 32.88 years (SD=12.21). Of these, 66.5% had a Charlson Comorbidity index (CCI) score of 0 (no comorbidities) and 67.3% were female. The average total medical costs were US$34 136 (median=US$12 691) annually, and SCD accounted for 60% of the total costs (mean=US$20 206, median=US$1204). Patients with >3 episodes had the highest annual SCD-related costs (mean=US$58 950) across all settings. Health care resource utilization (HCRU) and costs increased substantially as the number of VOC episodes increased. This study was limited to observation of associations rather than causal inference, and by possible coding and identification discrepancies and the restricted generalizability of the population.

**Conclusions:**

VOC has a severe impact on medical resource use and costs among the adult SCD population. Further research among broader study populations is needed to facilitate the reduction of VOC episodes and thereby improve clinical and economic outcomes for SCD patients.

## INTRODUCTION

Sickle cell disease (SCD) is a complex genetic blood disease in which erythrocytes have the propensity to change into a crescent (sickle) shape. This increases interactions with other endovascular cells and causes endothelium dysfunction, inflammation, and vascular damage.^1^,^2^ Of note, multi-cell adhesion between red blood cells, white blood cells, platelets, and endothelial cells can result in painful vaso-occlusion.^3^–^5^

Vaso-occlusive crisis (VOC) is the hallmark of SCD and sometimes the precursor to many life-threatening complications such as acute chest syndrome (ACS), stroke, splenic sequestration, and multisystem organ failure.^6^,^7^ Although VOC is painful and often requires immediate medical intervention such as emergency care and hospitalization, studies have shown that VOC episodes are also sometimes managed at home without emergency department (ER) visits or hospitalization.^8^,^9^ As VOCs significantly impact SCD patients’ quality of life (QOL), it is important for clinicians to minimize their number.

In addition to its serious impact on QOL, SCD and its complications incur a significant economic burden. It is estimated that in 2005, the mean annual expenditure for children with SCD was US$11 702 under Medicaid coverage and US$14 772 under employer-sponsored insurance.^10^ In particular, SCD-related health care costs for adults rise because many are forced to rely on urgent care, as their access to preventive and comprehensive health care is limited by a lack of providers and reduced insurance coverage.^11^ In one study on costs between 2001–2005, the per patient-month total health care costs of patients aged 50–64 years averaged US$2562—three times that of a group aged 0–9 years.^12^

SCD management is complicated as well as costly, in part due to the many dimensions of care. For instance, prevention of infectious diseases and SCD-related complications is necessary from early childhood. Such preventive approaches include newborn screening, anti-infection vaccinations, and the use of antibiotics from birth until completion of the initial series of vaccinations for encapsulated organisms. Also, to preempt the onset of neurologic complications, annual transcranial doppler ultrasonography screening is used to monitor stroke risk and assess the need for prophylactic transfusion therapy to prevent primary strokes.^13^

Among SCD maintenance treatments, blood marrow transplant (BMT), the amino acid L-glutamine (Endari^™^), voxelotor, and crizanlizumab-tmca have recently been added to the established use of blood transfusion and hydroxyurea (HU). HU has been shown to reduce the incidence of VOC and ACS as well as decrease necessary blood cell transfusions and overall health care resource utilization (HCRU); nonetheless, it remains underutilized.^14^–^18^

Because of these high levels of service and resource requirements, one of the main challenges of SCD management is HCRU, which is driven primarily by costly ER visits and hospitalizations.^19^ As its onset is often sudden and severe, VOC routinely requires urgent interventions (such as hospital admissions and emergency care) to prevent further life-threatening complications.^13^,^20^ Together with its contribution to morbidity and mortality, VOC’s substantial elevation of HCRU and costs has made VOC management an emphasis of SCD treatment patterns.^21^

As there is limited evidence on the economic burden of SCD, especially for adult patients, the purpose of this study was to provide a comprehensive breakdown of adult SCD patient direct medical costs, including all-cause and SCD-related HCRU and costs. Specifically, we stratified total costs (by the number of VOC episodes) and costs per VOC episode (by care setting) to provide a detailed examination of the association between VOC frequency and SCD economic burden.

## METHODS

### Study Design

This was a retrospective cohort analysis of patient-level data extracted from the United States Medicaid MAX databases from January 1, 2009 through December 31, 2013. The most recent 5 years of data were used at the time of the study and data was accessed through the Centers for Medicare and Medicaid Services (CMS).

The study included fee-for-service (FFS) patients from all available states and Managed Care enrollees who resided in the following 14 states with complete data: Arizona, California, Indiana, Kansas, Kentucky, Minnesota, Nebraska, New Jersey, New Mexico, New York, Oregon, Tennessee, Texas, and Virginia (managed care information for patients residing elsewhere was incomplete in the CMS MAX database).^22^

### Patient Selection

Patients were included in the study if they were aged ≥18 years and had an SCD diagnosis in any position (ICD-9-CM codes 282.41, 282.42, 282.60–282.69) during the identification period (July 1, 2009–December 31, 2012). The date of the first observed SCD-related diagnosis during the identification period was designated as the index date. Patients were required to have continuous enrollment with medical and pharmacy coverage for the 6-month baseline and ≥1 year of follow-up after the index date, and were followed until the earliest of disenrollment, death, or end of study. Patients who were enrolled in a clinical trial during the study period were excluded due to unique treatment patterns that could influence study outcomes.

Patients with VOC episodes were identified at any time after the index date using ICD-9-CM codes (282.42, 282.62, 282.64, 282.69). Because treatments for 1 VOC episode can be administered in multiple settings within a given time window, discrete VOC claims within a 3-day gap were combined and recorded as a single VOC episode.

## OUTCOME ASSESSMENTS

### HCRU and Costs For 1 Year

Annual all-cause and SCD-related HCRU and costs for each patient were evaluated during the 12 months after the index date. For HCRU analysis, the length of inpatient stays and the number of visits were computed for the fixed 1-year period (including inpatient, outpatient, ER, outpatient hospital, ambulatory surgery center, lab, hospice, longterm care [LTC], and pharmacy department visits).

For analysis of health care costs by facility type, only FFS patients were calculated (Managed Care cost data are unavailable in the CMS MAX database). Costs were adjusted to 2013 US dollars using the medical care component of the Consumer Price Index (CPI).

HCRU and costs were considered SCD-related if they were associated with an SCD diagnosis, SCD medications (eg, opioids, nonsteroidal anti-inflammatory drugs [NSAIDs], and hydroxyurea), or other SCD management procedures (eg, blood transfusion).

SCD-related HCRU and costs were also stratified by number of VOC episodes in the 1-year follow-up period. Patients were separated into four cohorts, according to number of VOC episodes (0, 1, 2, and ≥3).

### Costs Per VOC Episode

Costs per VOC episode were identified in any setting and classified by place of service using a hierarchical order: inpatient, ER, outpatient, office, and other. The definition of outpatient VOCs included those in the outpatient hospital, ambulatory surgery center, nursing facility, or long-term care settings. Office VOCs included those in the independent clinic, federally qualified health center, state or local public health clinic, or rural health clinic settings.

Health care costs during the VOC episodes were identified and included the total medical costs in any setting and outpatient pharmacy costs related to SCD treatments. Average costs per VOC episode were calculated for all VOC episodes and for each setting of care.

### Statistical Analyses

Numbers and percentages are reported for categorical variables. Mean values and standard deviations are reported for continuous variables.

## RESULTS

### Patient Baseline Characteristics

**Figure 1:**
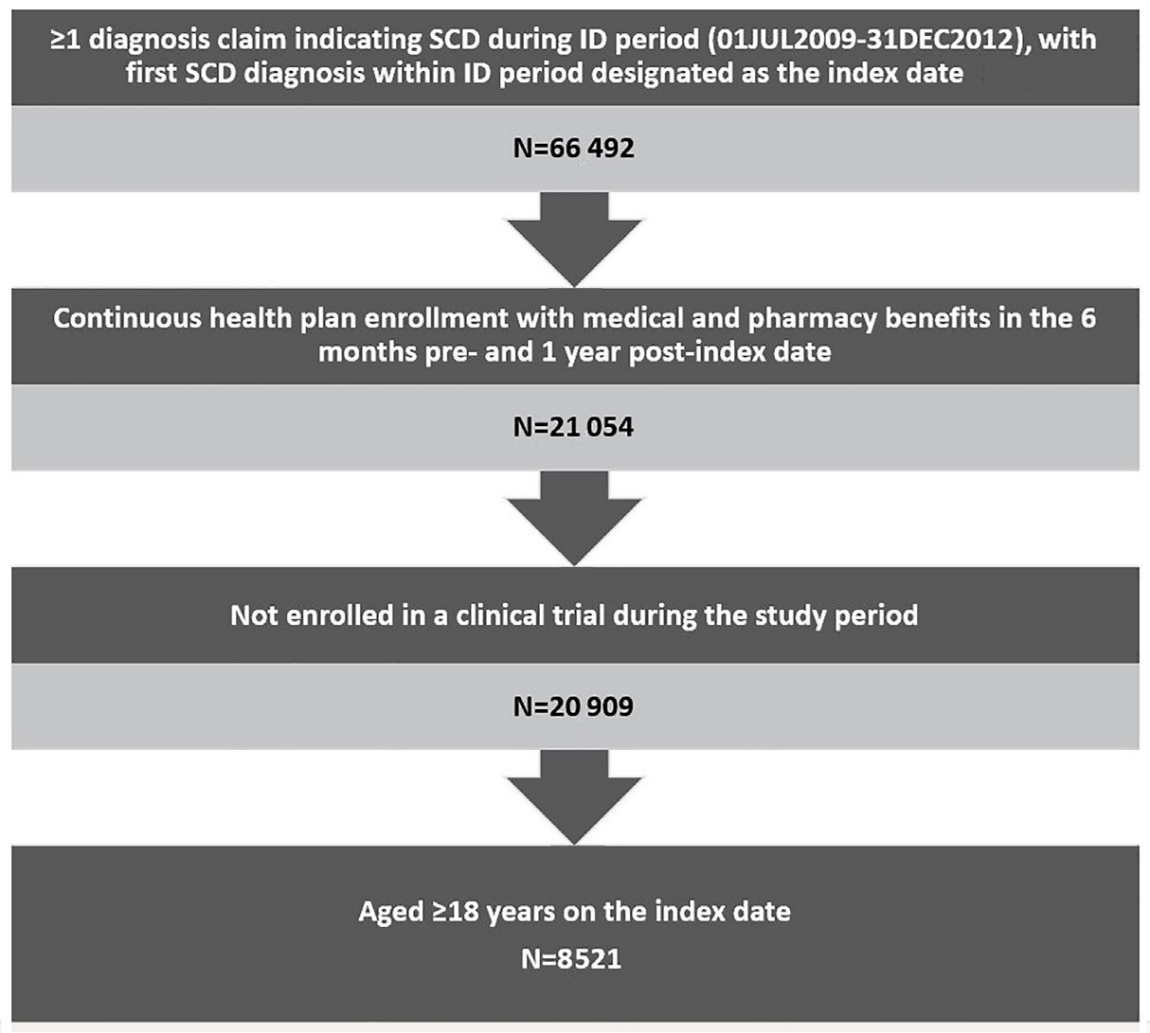
Patient Selection ID: identification; SCD: sickle cell disease

This analysis included 8521 qualifying adult patients with SCD (Figure 1), most of whom were relatively young African American women with no comorbidities. The mean age of patients was 32.9 years (SD=12.2) and more than half (51.9%) were in the 18–30 year age group. Nearly three-quarters (74%) were African American, followed by Hispanic (10.1%) and unknown race (6.8%). Females accounted for 67.3% of the patients and 66.5% had a CCI score of 0. (Table 1)

Among all eligible patients, 24.6% had ≥1 VOC requiring an inpatient stay during the 6-month baseline period. Infectious disease (19.74%) was the most common baseline comorbidity, followed by asthma (10.9%), fever (9.3%), and neoplasms (7.2%). (Table 2)

For baseline SCD management, 44.8% of the patients were prescribed antibiotics, followed by acetaminophen (44.1%), folic acid (29.8%), opioids (28.1%), and NSAIDs (26%); 10.8% used hydroxyurea, and 14% had a blood transfusion. Assessment of baseline all-cause HCRU found 53.6% of the patients had ≥1 outpatient ER visit and 29.8% had ≥1 inpatient visit. (Table 2)

### Annual All-Cause and SCD-Related HCRU and Costs During the Post-index Period

Among the 8521 total adult patients with SCD, 49.8% (N = 4247) had an FFS Medicaid plan and were included in the analysis for health care costs. The average total medical costs were US$34 136 (median=US$12 691) in 1 year and SCD accounted for 60% of the total costs (mean=US$20 206, median=US$1204). The largest all-cause medical cost driver was inpatient costs, with average annual expenditure at US$17 535 (median=US$1590) per patient; SCD accounted for around 87% of these inpatient costs (mean=US$15 237). SCD also contributed to high proportions of costs in other settings such as pharmacy (67%) and ER (68%).

The study population also utilized health care resources frequently. Almost all the enrolled patients had ≥1 all-cause pharmacy visit (95.2%), and 86% of these visits were related to SCD treatments. Large proportions of the patients had ≥1 all-cause outpatient hospital (87.4%) and outpatient office visit (83.6%), with 57% and 75% of these visits due to SCD, respectively. (Table 3)

### SCD-Related Costs and HCRU Stratified by Number of VOC Episodes

Among the 8521 enrollees, most had no VOC episode during the 1-year follow-up period, but a large number had ≥3 episodes. Specifically, 4452 patients had no episode, 1253 had 1 episode, 570 had 2 episodes, and 2246 had ≥3 episodes.

Regarding SCD-related HCRU, patients with ≥3 episodes had the highest mean number of outpatient hospital visits (mean=33.25; SD=37.02), outpatient ER visits (mean=9.12; SD=15.35), inpatient visists (mean=4.72; SD=5.73), inpatient length of stay (mean=26.11; SD=35.76), and pharmacy visits (mean=3.24; SD=1.26). As expected, utilizations increased in positive correlation with the number of episodes (eg, patients with ≥3 episodes had four times more inpatient visits than patients with 2 episodes). (Figure 2A)

Evaluation of SCD-related costs found total costs were highest among patients who had ≥3 episodes, with a mean of US$58 950 (SD=US$93 147). Across the 4 cohorts, the largest cost driver was inpatient costs; the ≥3 cohort had the highest mean inpatient costs of US$45 088 (SD=US$85 112), followed by pharmacy, outpatient hospital, and outpatient ER costs. As with utilizations, costs increased for patients with a higher number of VOC episodes (eg, patients with ≥3 episodes incurred 4–5 times the total costs and inpatient costs of the 2-episode cohort). (Figure 2B)

**Figure 3:**
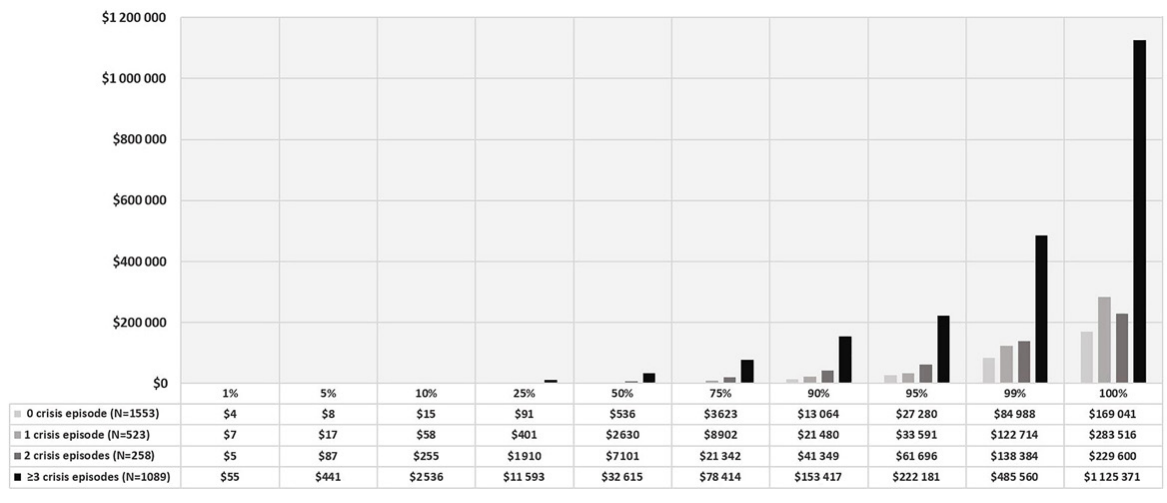
Frequency Distribution for Total Healthcare Cost Among FFS SCD Patients Stratified by Number of Crisis Episodes.

In addition, the frequency distribution of SCD-related health care costs for patients with FFS coverage was examined. Patients with total cost of US$0 were excluded because the actual cost might not have been captured. The mean total cost for patients with 0 pain crisis to ≥3 crisis ranged from US$5516 to US$62 577 during the 12 months of follow-up. Also, the frequency distribution for the total SCD-related health care costs also showed that patients with ≥3 crisis incurred a maximum total health care cost of US$1 125 371. (Figure 3)

### Costs Per VOC Episode by Different Places of Service

Among the 4247 total adult SCD patients with FFS Medicaid, 2316 (50.9%) had ≥1 VOC episode after the index date. A total of 40 772 VOC episodes were identified in the following settings (in hierarchical order): 15 395 from inpatient, 14 779 from ER, 7420 from outpatient, 2365 from office, and 813 from other settings. The mean duration of each episode was 5.5 days (SD=11).

**Figure 4:**
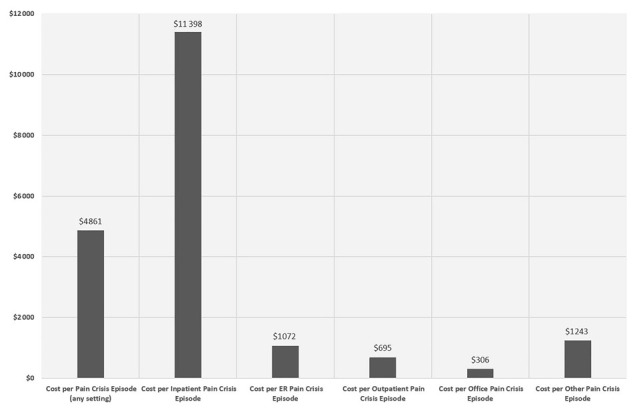
Healthcare Costs per VOC Episode by Settings ER: emergency room

The mean total medical cost per VOC episode was US$4861 (SD=US$14 296). The mean costs per VOC episode varied widely across different settings, with inpatient VOCs incurring by far the highest per-episode cost (mean=US$11 398, SD=US$21 358), followed by ER (US$1072 [SD=US$3458]), outpatient (US$695 [SD=US$2437]), and office settings (US$306 [SD=US$1480]). (Figure 4)

## DISCUSSION

This study explored the medical expenditures of adult SCD patients, detailing the breakdown of utilizations and costs not only by number of VOC episodes but also per-episode, stratified by setting. The results revealed the biggest cost drivers to be inpatient vistis and pharmacy, and shed more light on the economic burden of the disease. Although other studies have explored the economic burden of SCD, most were conducted at the individual state level and relevant only to specific treatments,^23^–^25^ or focused mainly on outcomes in the pediatric population.^10^,^26^,^27^ This study adds real-world evidence with more granular insight into clinical profiles and economic outcomes among the adult SCD population at the national level.

Previous studies indicate that VOC frequency, more so than pain duration or pain severity, is associated with increased utilization of health services,^28^ and the economic burden of SCD has been recognized for decades. Multiple studies have reported substantial medical expenditures among various SCD populations, with the average annual adult SCD-related costs ranging from US$11 508–US$21 792 in 2005,^29^–^31^ which is consistent with this study’s finding of US$20 206.

In addition, the present study showed that VOC episodes were the primary reason for the high medical utilizations and costs among the study population, based on the findings on the ratio of HCRU and costs, stratified by different number of VOC episodes. As expected, the study showed that patients with the highest VOC frequency (the ≥3 episode cohort) incurred the highest health care costs. They accounted for 26% of the total sample, indicating that a large group of adult SCD patients were suffering from high-frequency VOC episodes, and this complication was inflating overall costs for the SCD population at large.

Hence, this study confirmed a correlation between episode frequency and HCRU and costs (the ≥3 episode cohort incurred 4–5 times more total SCD-related medical costs and inpatient costs than the 2 episode cohort, for instance). This could reflect increases in episode severity requiring more resources, which was further suggested by the per-episode cost findings, as inpatient VOC episodes had high mean costs. Moreover, the costs per VOC episode included both VOCrelated costs and all other medical costs incurred by patients with VOC during the episode period, which more precisely reflects the real-world medical costs associated with VOCs.

It is also important to note there are some barriers associated with acute VOC management. These include incomplete understanding of the underlying mechanism of vaso-occlusive pathophysiology, sparse access to SCD experts, lack of evidence-based treatment guidelines, and inconsistent adherence to health care provider indications.^32^ As VOC has a significant impact on patients and their families, the factors that contribute to VOC management should be re-evaluated and brought to the table for health care experts and decision makers to move toward more effective interventions. Also, medication like the newly approved crizanlizumab-tmca, which has shown efficacy in the reduction of VOC frequency in SCD patients, could be included in the management of SCD patients.^33^ This may help alleviate the burden of frequent VOCs in SCD patients.

Further, an adjunct analysis examined the frequency distribution for the total SCD-related health care costs for FFS SCD patients. This analysis showed that patients with frequent crises could be associated with a huge economic burden of over US$1 million annually, as observed in some patients with more than 3 crises in a year. However, we cannot ascertain whether this high cost is mainly because of the high number of crises or the severe complications associated with the crises.

### Limitations

While the study findings elucidate medical resource use and expenditures in this population, several limitations should be noted.

The study relied on descriptive statistical analysis of outcomes, which requires caution in drawing conclusions from cohort comparisons, as these results are trends without direct experimental testing, such as randomized controlled trials, and therefore imply only correlation, not causation. Moreover, claims data analysis has its own limitations. For example, the data and corresponding outcomes may be affected by administrative diagnosis coding errors, codes entered as rule-out criteria and not actual indications of disease presence, and deliberate misdiagnoses to justify claims for off-label prescriptions.

Another limitation of the study is that in order to capture all SCD patients including those with low health care utilizations, patients with ≥1 SCD diagnosis in any setting were identified as SCD patients; this might have inadvertently included some non-SCD patients due to coding discrepancies. Although this study shows a large proportion of patients had high VOC frequency, we believe there is underreporting of the true number of SCD patients who experienced VOC and frequency of the VOC because home-managed VOCs were not capturable in the dataset used for this study.

In addition, the dataset was from 2009–2013 and real-world clinical management of SCD may have changed since the end of the study period.

The dataset also presented another limitation specific to this study. As the target was HCRU and costs by facilities, only FFS patients were included in the analysis because costs for Managed Care enrollees are not recorded in the Medicaid MAX dataset. This reduced the sample size and limited the generalizability of the study outcomes to other populations.

Notwithstanding these limitations, the study does lay the groundwork for further research that can put the analysis into more specific statistical models and examine how the economic outcomes are related to the reduction of VOC episodes, controlling for covariates such as patient demographics and clinical characteristics.

## CONCLUSIONS

The study found VOC episodes are associated with significant economic burden among patients with SCD, as they led to higher frequencies of inpatient utilizations, which were the largest cost driver among the study population. Moreover, HCRU and costs were closely and positively associated with the number of VOC episodes. In particular, among patients who had 3 or more VOC episodes per year, those episodes accounted for over 90% of the SCD-related inpatient visits and costs. Thus, VOC frequency can be seen as a strong indicator for medical resource use and expenditures. These findings warrant future investigations among broader study populations with more sensitive analysis, and ultimately may help clinicians reduce VOCs and thereby mitigate the overall clinical and economic burden of SCD.

## Figures and Tables

**Figure 2a:**
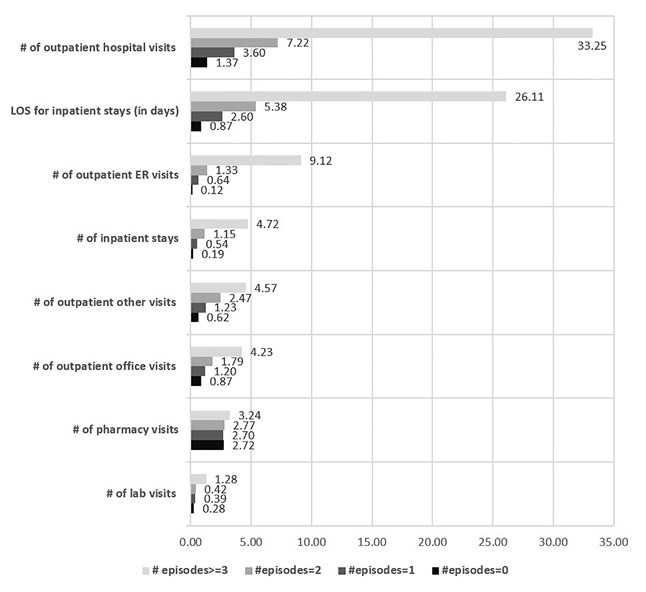
SCD-related HCRU Stratified by Number of Pain Episodes in 1 Year ER: emergency department; LOS: length of stay

**Figure 2b:**
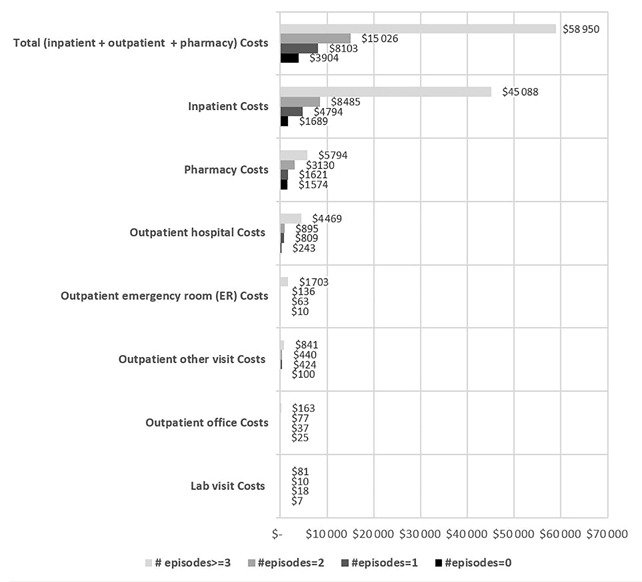
SCD-related Medical Costs Stratified by Number of Pain Episodes in 1 Year ER: emergency department; LOS: length of stay

**Table 1:** Patient Demographic Characteristics

	Sickle Cell Patients* (Age ≥18) (N = 8521)	
Baseline Demographics	N/Mean	%/SD
**Age (Years)**	32.88	12.21
**Age Group**		
18–30	4421	51.9%
31–45	2521	29.6%
46–64	1526	17.9%
65+	53	0.6%
**Sex**		
Male	2790	32.7%
Female	5731	67.3%
**Race/Ethnicity**		
White	534	6.3%
Black	6305	74%
Hispanic	862	10.1%
Other	245	2.9%
Unknown	575	6.6%
**Geographic Region**		
Northeast	3800	44.6%
South	2266	26.6%
West	1612	18.9%
North Central	843	9.9%

* The following ICD-9-CM codes 282.41, 282.42, 282.60–282.69 were used to identify SCD patients.

Abbreviations: N, number; SD, standard deviation.

**Table 2:** Baseline Clinical Characteristics and Utilization

	Sickle Cell Patients (Age ≥18)	
	(N = 8521)	
Baseline Clinical Characteristics	N/Mean	%/SD
**Charlson Comorbidity Index (CCI) Score**	0.65	1.25
0	5665	66.5%
1	1593	18.7%
2–3	941	11.0%
4+	322	3.8%
**Patients With Baseline VOC Episodes**	2097	24.6%
**Baseline Number of VOC Episodes (6 months)**	1.04	2.72
Individual Comorbid Conditions*		
Neoplasms benign and malignant	617	7.2%
Seizures	393	4.6%
Asthma	928	10.9%
Upper respiratory tract infections	565	6.6%
Acute chest syndrome	233	2.7%
Infectious and parasitic diseases	1682	19.7%
Fever	792	9.3%
Constipation	501	5.9%
Chronic pain	418	4.9%
Iron overload	367	4.3%
Aseptic (avascular) bone necrosis	359	4.2%
**Baseline SCD Management**		
SCD Medication*		
Antibiotics	3819	44.8%
Acetaminophen	3756	44.1%
Folic acid	2542	29.8%
Opioids (narcotics)	2393	28.1%
NSAIDs	2214	26.0%
Hydroxyurea	921	10.8%
Other SCD Management*		
Blood transfusions	1189	14.0%
Baseline All-Cause Health Care Resource*		
Any pharmacy visit	7266	85.3%
Any outpatient hospital visit	5992	70.3%
Any outpatient office visit	5923	69.5%
Any outpatient ER visit	4563	53.6%
Any outpatient other visit	3580	42.0%
Any lab visit	3318	38.9%
Any inpatient stay	2543	29.8%

* Only individual comorbidities with ≥4% are reported.

Abbreviations: ER, Emergency room; N, number; NSAIDs, nonsteroidal anti-inflammatory drugs;

PDE-5, phosphodiesterase type 5; SD, standard deviation; VOC, vaso-occlusive crisis.

**Table 3:** Annual All-Cause and SCD-Related HCRU and Costs

Follow-Up All-Cause HCRU and Costs in the First 12 Months			
Facility Type	% Patients With ≥1 Visit	Mean Costs	Median of Costs
**Inpatient**	56.3%	US$17 535	US$1590
**Outpatient other**	56.0%	US$6891	US$0
**Pharmacy**	95.2%	US$3898	US$0
**Long-term care (LTC)**	3.0%	US$2665	US$169
**Outpatient hospital**	87.4%	US$2081	US$0
**Outpatient ER**	73.3%	US$592	US$0
**Outpatient office**	83.6%	US$397	US$0
**Hospice**	0.1%	US$34	US$3577
**Lab**	55.5%	US$33	US$0
**Ambulatory surgery center**	4.5%	US$12	US$281
**Total (inpatient + outpatient + LTC + pharmacy) Cost**		US$34 136	US$12 691
**Follow-Up SCD-Related HCRU and Costs in the First 12 Months**			
**Facility Type**	**% Patients with** ≥**1 visit**	**Mean Costs**	**Median of Costs**
**Inpatient**	42.7%	US$15 237	US$0
**Pharmacy**	82.0%	US$2584	US$0
**Outpatient hospital**	65.2%	US$723	US$0
**Outpatient other**	28.7%	US$685	US$0
**Long-term Care (LTC)**	1.2%	US$498	US$0
**Outpatient ER**	49.9%	US$402	US$0
**Outpatient office**	47.7%	US$64	US$0
**Lab**	27.1%	US$8	US$0
**Ambulatory surgery center**	1.7%	US$5	US$0
**Hospice**	0.0%	US$0	US$0
**Total (inpatient + outpatient + LTC + pharmacy) Cost**		US$20 206	US$1204

Note: Health care costs were examined among patients with FFS Medicaid plan.

Abbreviations: LTC, long-term care; SCD, sickle cell disease; HCRU, health care resource utilization; FFS, fee-for-service; ER, emergency department
